# Early and midterm results of radiofrequency ablation (Rafaelo^®^ procedure) for third-degree haemorrhoids: a prospective, two-centre study

**DOI:** 10.1007/s10151-022-02608-x

**Published:** 2022-03-19

**Authors:** S. Tolksdorf, D. Tübergen, C. Vivaldi, M. Pisek, F. Klug, M. Kemmerling, H. Schäfer

**Affiliations:** 1 Proctology and Endoscopy Practice, Pan Clinic, Zeppelinstr. 1, 50937 Cologne, Germany; 2 Coloproctology Practice, Münster, Germany; 3 Clinic for General, Visceral and Tumour Surgery, University of Cologne, Cologne, Germany

**Keywords:** Radiofrequency ablation, RFA, Prolapsing haemorrhoids, Minimally invasive treatment, Outpatient procedure

## Abstract

**Background:**

The aim of this study was to evaluate the safety and efficacy of radiofrequency ablation (RFA) for treating third degree haemorrhoids, with a follow-up over 2 years.

**Methods:**

We conducted a prospective, two-centre study to assess RFA of third-degree haemorrhoids in an outpatient setting. Treatment was performed under local anaesthesia, optionally in combination with sedation. The primary endpoint was analysis of a proctological symptom score ([PSS] bleeding, itching, pain, soiling) and proctological examination to detect recurrence at 1, 6, 12 and 24 months after surgery. The secondary endpoints were postoperative complications, incidence of postoperative pain, including administration of analgesics and time to return to daily routine.

**Results:**

Ninety-eight patients were included in the study. The mean age of the patients was 49.1 ± 10.9 (mean ± SD). 83 patients (84.7%) were male and 15 patients (15.3%) were female. The follow-up involved 100% (1 month), 95% (6 months), 86% (12 months) and 74% after 24 months. The individual symptom scores and overall PSS score decreased significantly in comparison to the initial score at each time point assessed. Prolapsed haemorrhoids decreased in comparison to the initial situation (100%) to 7.2% (1 month), 3.5% (6 months), 13.1% (12 months) and 13.7% (after 24 months). Thirteen patients (12.7%) required repeat haemorrhoid therapy during the 2-year follow-up period. The mean maximum pain score after the procedure was 2.5 ± 2.7 (determined with the visual analogue scale), while 33 (33.7%) patients reported having no pain. 59 (60.2%) patients did not take analgesics after the procedure. Eleven patients (11.2%) experienced minor complications (bleeding, fever, cramps, diarrhoea, anal venous thrombosis) but did not require additional treatment. Eight cases (8.2%) of major complications (infection, bleeding, severe pain) required treatment with antibiotics, a second intervention, analgesics or hospitalization.

**Conclusions:**

RFA is safe and effective for treatment of third-degree haemorrhoids. The main advantages of this new method are its use on an outpatient basis under local anaesthesia, a very low level of postoperative pain and significant control of haemorrhoid symptoms over 2 years.

## Introduction

Patients with haemorrhoidal disease often avoid visiting a physician, not only because the anal area is part of their intimate region, but because they fear the possible treatment could be painful.

Patients with haemorrhoidal disease suffer from itching, bleeding, oozing and pain that can seriously impact their quality of life. Internal haemorrhoids can be treated successfully with infrared coagulation, sclerotherapy and/or rubber band ligature in combination with a high-fibre diet, while external haemorrhoids are mostly treated surgically [1].

Classic excision of prolapsing haemorrhoids is effective but usually associated with significant postoperative pain and a higher percentage of patients being unable to work for some time after surgery. To avoid general anaesthesia, a hospital stay and significant postoperative pain in the treatment of prolapsing haemorrhoids, we introduced radiofrequency ablation (RFA) under local anaesthesia as an outpatient procedure. The use of a probe, with its tip located directly in the haemorrhoid, reduces intraoperative and postoperative trauma. Our first study with 100 patients yielded encouraging results around the safety of the method and the reduction of haemorrhoid symptoms [2]. The aim of the present study was to assess the long-term efficacy of this innovative method in third degree haemorrhoid disease using a validated proctological score system (PSS) [3]. We also documented complications caused by the procedure, postoperative pain (visual analogue scale [VAS] score), postoperative analgesic use and time until return to daily routine.

## Materials and methods

We conducted a prospective, two-centre study with 5 specialised coloproctologists (Proctology and Endoscopy Practice, Cologne, Germany; Coloproctology Practice, Münster, Germany) who have been treated patients RFA since 2015. Each surgeon attended a workshop on RFA treatment and had treated at least 5 patients before participating in the study, so they were familiar with the use of RFA. The study was approved by the Ethics Committee of the University of Cologne, Germany. We prospectively included patients with symptomatic third-degree haemorrhoidal disease in a maximum of two segments from September 2016 to May 2018. Diagnosis was made by examination and proctoscopy.

The following patients were excluded: patients with circumferential anal mucosal prolapse, first, second- and fourth-degree haemorrhoids, third degree haemorrhoids with more than 2 piles, pregnant or breastfeeding patients, patients with pacemakers, patients taking anticoagulant medication (except for acetylsalicylic acid 100 mg) and patients with hypercoagulopathy, a chronic inflammatory disease or anorectal cancer.

**Fig. 1:**
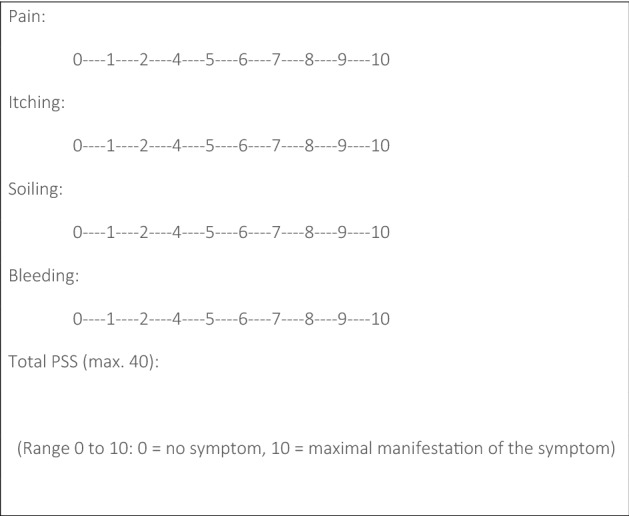
The proctological symptom scale (PSS)

Age, sex, body mass index (BMI) and previous haemorrhoid treatment were recorded before the procedure. Haemorrhoid stage was determined during preoperative proctological examination. Stage 3 haemorrhoids were diagnosed when the patient reported the need for manual replacement and when haemorrhoidal tissue became visible on the outside spontaneously or after the patient pressed. Symptoms of faecal incontinence were recorded and symptoms of soiling/oozing were attributed to the haemorrhoidal disease. Haemorrhoid symptoms were recorded using the proctological score system (PSS, Fig. 1) [3].

Patients had to give informed consent for the procedure and prospective study. The procedure was carried out in local anaesthesia. On request, we performed the procedure under sedation with propofol plus local anaesthesia in 15 cases (15.3%).

**Fig. 2:**
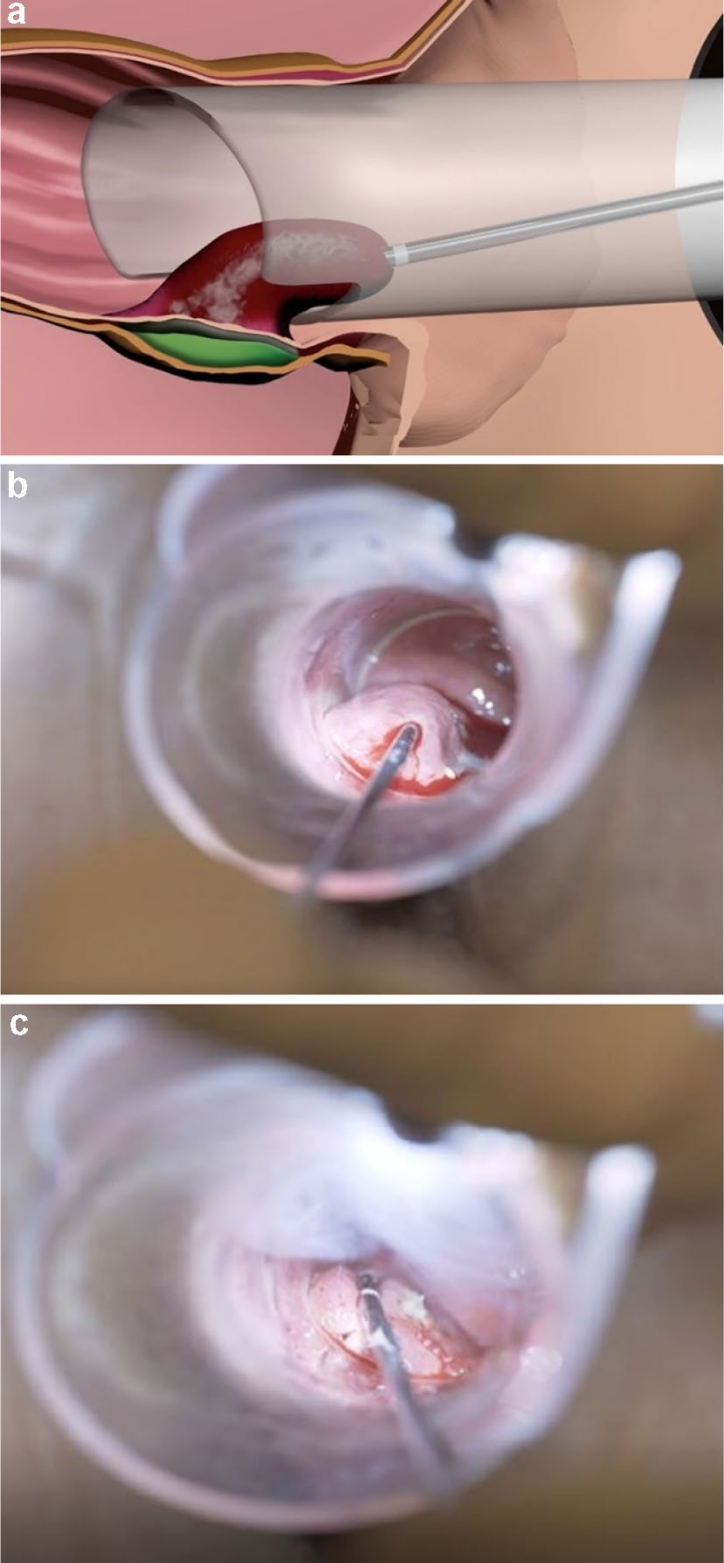
**a** Schematic overview of the Rafaelo^®^ Procedure (picture kindly provided by FCare Systems), **b** Areal-time image of the Rafaelo^®^ Procedure. The tip of the probe has been inserted into the haemorrhoid. **c** Coagulation of the haemorrhoid surface

The procedures were performed with the patient in the lithotomy position. After the haemorrhoid node was located with a proctoscope, a local anaesthetic (3–6 ml 1% lidocaine solution) was injected between the submucosa and muscle layer to prevent thermal damage to the muscle layer. The tip of the probe was inserted above the dentate line along the haemorrhoid tissue towards the upper pole (Fig. 2a, b).

Radiofrequency energy was applied in four positions with a maximum of 2500 Joules per node. During the procedures, patients were asked for provide feedback on whether they felt warmth. If the feeling of heat became painful, the procedure was interrupted and the tissue was cooled with ice-cold gauze. The probe was removed if lightening of the tissue colour demonstrated effective heat damage. The puncture site was coagulated separately. Finally, the haemorrhoid surface was coagulated in several points to promote haemorrhoid retraction by mucosal coagulation (Fig. 2c). The final step was to control bleeding and tissue was cooled again. The same procedure was repeated if a second haemorrhoid that might be present.

Patients left the coloproctologist’s office immediately after the procedure or after they had recovered from sedation. For outpatient care, analgesics such as ibuprofen (max. 1200 mg per day) were prescribed.

Follow-up examinations took place in the coloproctologist’s office on days 7 and 28, as well as after 6, 12 and 24 months.

Proctoscopy was carried out on day 28 and again after 6, 12 and 24 months.

### Statistical analysis

The data were analysed using SAS Version 9.4 (SAS Institute Inc., Cary, NC, USA). All statistical analyses were descriptive and exploratory in nature.

Summary statistics for continuous variables include mean with two-sided 95% confidence interval (CI), standard deviation (SD) or standard error (SE).

For categorical variables, the number (n) and proportion of patients (%) are presented.

For inference statistics, the paired t test and the two-group t test were used to analyze continuous PSS score values. The binomial test was used to analyze categorical prolapse data.

## Results

Between September 2016 and May 2018, 98 patients were treated using RFA. Demographic data are shown in Table 1. Neither the patients’ medical history nor the proctological examinations revealed faecal incontinence before RFA.

**Table 1:** Demographic data

Age (years)	Mean ± SD	49.1 ± 10.9
Sex		49.1 ± 10.9
Male	*n* (%)	83 (84.7)
Female	*n* (%)	15 (15.3)
BMI (kg/m^2^)	Mean ± SD	25.3 ± 4.0
Previous treatments	n (%)	57 (58.2)
Ligation therapy and/or sclerotherapy	*n* (%)	29 (29.6)
Longo-operation	*n* (%)	3 (3.1)
Milligan-Morgan excision	*n* (%)	1 (1.0)
RFA	*n* (%)	13 (13.3)
Unknown	*n* (%)	11 (11.2)
Joules	Mean ± SD	3006 ± 1186
Anaesthesia		
Local anaesthesia	*n* (%)	83 (84.7)
Sedation + local anaesthesia	*n* (%)	15 (15.3)

*RFA* radiofrequency ablation, *BMI* body mass index

Ninety-three (94.9%) patients had a follow-up examination after 6 months, 84 (85.7%) patients after 12 months and 73 (74.4%) patients 24 months after the intervention. Eighty-three (84.7%) patients were treated under local anaesthesia, while 15 (15.3%) patients received a sedative plus local anaesthesia (mostly propofol) at their own request, which was given during the procedure. Fifty-eight (59.2%) patients were treated for a single haemorrhoid, while 40 (40.8%) were treated for at least 2 haemorrhoids at different sites. On average, 3029 Joules were administered per intervention. The mean duration of ablation of each treated pile was 2:07 ± 1:27 min (range 0: 28–4:17 min).

Complications occurred during or after the intervention in 19 (19.4%) cases (Table 2). There were 11 (11.2%) minor complications. Two patients experienced slight bleeding that did not require any intervention. In addition, 2 patients reported transient fever or chills that did not require additional treatment. Two patients complained of temporary constipation or diarrhea. No additional treatment was required in these patients. One patient described painful anal cramps after RFA that resolved spontaneously. Three patients developed anal vein thrombosis after the procedure and were treated conservatively with an ointment. Shortly after RFA 1 patient complained of increased urinary urgency, which disappeared without specific treatment.

**Table 2:** Complications

	*n* (%)
Total	19 (19.4)
Minor	11 (11.2)
Light bleeding	2 (2.0)
Sub-febrile temperatures/cold	2 (2.0)
Meteorism/diarrhoea	2 (2.0)
Pain/cramping	1 (1.0)
Swelling/anal vein thrombosis	3 (3.1)
Increased urination	1 (1.0)
Major	8 (8.2)
Bleeding	4 (4.1)
Infected necroses/ulcers	2 (2.0)
Severe pain	2 (2.0)

There were 8 major complications. Two patients had bleeding at the injection site, which had to be sutured during the procedure. Two patients with significant anal bleeding 5 and 7 days after RFA underwent diagnostic rectoscopy in the operation theatre, but no active bleeding was detected. One patient developed a chronic pain syndrome due to an ulcer at the former site of the haemorrhoid. This was successfully treated with a subsequent Milligan-Morgan operation. One patient developed a haemorrhoidal ulcer/necrosis with pain during the first week; this was successfully treated with antibiotics. Two other patients reported severe pain, which was successfully treated with analgesics.

**Fig. 3:**
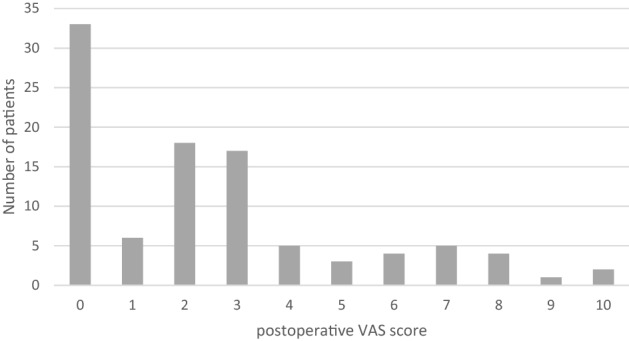
A graph showing the postoperative VAS score. *VAS* visual analogue scale

**Fig. 4:**
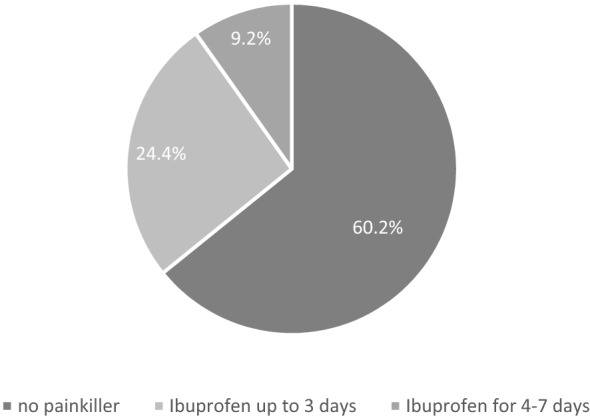
A graph showing the use of analgesics during the postoperative course

Thirty-three (33.7%) patients reported that they did not experience any pain in the first days after treatment (Fig. 3). Fifty-nine patients (60.2%) did not require any analgesics (Fig. 4).

Seventeen patients (17.3%) required a certificate of inability to work for a period of 1–11 days (mean 3.3 days). The remaining patients continued with their daily activities and reported that RFA did not result in restrictions in their daily life.

**Fig. 5:**
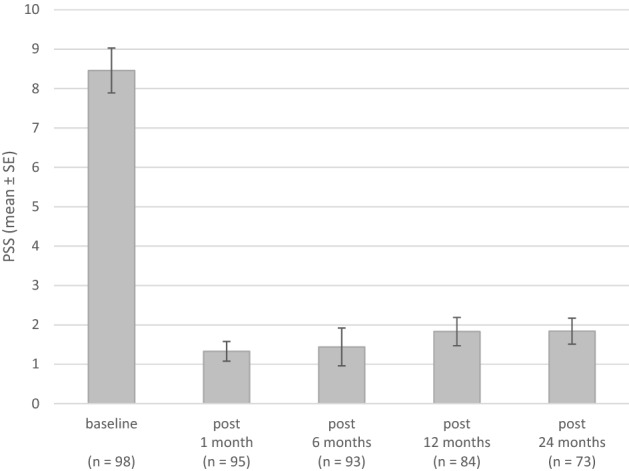
A graph showing the total proctological symptom score (PSS): change from baseline *p* < 0.001 (paired *t* test). *PSS* proctological symptom scale

Each individual symptom score and overall PSS score decreased significantly between the baseline and 24 months (Fig. 5; Table 3). The bleeding score and total PSS score registered an increase at 24 months. On univariate analysis there was no difference in the PSS score in relationship to age, gender, BMI, previous treatments, energy applied (Joules) and type of anesthesia (Table 4; data for 6 and 24 months are shown).

**Table 3:** Mean PSS score values at baseline and post-baseline

	*n*	Baseline				Post-baseline				Difference				*p* value paired *t* test
		95% CI			SD	95% CI			SD	95% CI			SD	
		Mean	(	)		Mean	(	)		Mean	(	)		
Baseline vs. post 1 month														
Total score	95	8.59	7.43	9.76	5.71	1.33	0.83	1.82	2.43	− 7.27	− 8.36	− 6.18	5.36	< 0.0001
Itching	95	1.32	0.90	1.74	2.05	0.37	0.17	0.57	0.99	− 0.95	− 1.35	− 0.56	1.93	< 0.0001
Pain	96	1.78	1.34	2.22	2.17	0.23	0.04	0.42	0.96	− 1.55	− 1.97	− 1.13	2.09	< 0.0001
Bleeding	96	3.31	2.71	3.92	2.98	0.35	0.12	0.59	1.16	− 2.96	− 3.54	− 2.38	2.86	< 0.0001
Oozing	96	2.14	1.62	2.65	2.56	0.36	0.13	0.60	1.18	− 1.77	− 2.26	− 1.28	2.43	< 0.0001
Baseline vs. post 6 months														
Total score	93	8.35	7.20	9.49	5.56	1.44	0.48	2.40	4.65	− 6.91	− 8.30	− 5.51	6.77	< 0.0001
Itching	93	1.37	0.94	1.80	2.08	0.37	0.09	0.64	1.33	− 1.01	− 1.46	− 0.55	2.21	< 0.0001
Pain	93	1.71	1.29	2.13	2.06	0.19	− 0.04	0.43	1.13	− 1.52	− 1.96	− 1.07	2.17	< 0.0001
Bleeding	93	3.19	2.59	3.80	2.95	0.42	0.14	0.70	1.37	− 2.77	− 3.41	− 2.14	3.08	< 0.0001
Oozing	93	2.08	1.55	2.60	2.57	0.46	0.16	0.76	1.46	− 1.61	− 2.21	− 1.02	2.88	< 0.0001
Baseline vs. post 12 months														
Total score	84	8.03	6.84	9.22	5.50	1.83	1.12	2.54	3.27	− 6.20	− 7.48	− 4.91	5.90	< 0.0001
Itching	84	1.38	0.92	1.83	2.12	0.52	0.23	0.82	1.37	− 0.85	− 1.31	− 0.39	2.12	0.0004
Pain	84	1.49	1.10	1.88	1.80	0.29	0.10	0.47	0.84	− 1.20	− 1.60	− 0.80	1.85	< 0.0001
Bleeding	84	3.08	2.44	3.72	2.95	0.62	0.28	0.96	1.57	− 2.46	− 3.10	− 1.83	2.91	< 0.0001
Oozing	84	2.08	1.52	2.65	2.62	0.40	0.14	0.67	1.23	− 1.68	− 2.31	− 1.05	2.90	< 0.0001
Baseline vs. post 24 months														
Total score	73	7.95	6.72	9.18	5.27	1.84	1.18	2.49	2.80	− 6.12	− 7.28	− 4.96	4.97	< 0.0001
Itching	73	1.38	0.89	1.87	2.10	0.25	0.11	0.38	0.57	− 1.13	− 1.62	− 0.64	2.09	< 0.0001
Pain	73	1.53	1.10	1.97	1.86	0.21	0.03	0.38	0.74	− 1.33	− 1.78	− 0.88	1.92	< 0.0001
Bleeding	73	3.08	2.37	3.80	3.07	0.88	0.47	1.28	1.75	− 2.21	− 2.90	− 1.51	2.97	< 0.0001
Oozing	73	1.96	1.36	2.56	2.58	0.51	0.16	0.85	1.47	− 1.45	− 1.99	− 0.91	2.32	< 0.0001

*PSS* proctological symptom score

**Table 4:** Subgroup analysis of overall PSS at 6 and 24 months

	6 months						24 months					
	95% CI					*p* value two-group *t* test	95% CI					*p* value two-group *t* test
	*n*	Mean	(	)	SD		*n*	Mean	(	)	SD	
Age												
≤ 40 years	20	1.45	0.21	2.69	2.65		15	2.40	0.46	4.34	3.50	
> 40 years	72	1.46	0.26	2.66	5.12	0.9922	58	1.69	1.00	2.38	2.61	0.4718
Gender												
Male	80	1.58	0.47	2.68	4.97		66	1.83	1.16	2.50	2.72	
Female	13	0.62	− 0.39	1.62	1.66	0.1890	7	1.86	− 1.62	5.34	3.76	0.9875
Previous haemorrhoid treatment												
Yes	45	1.36	0.47	2.24	2.93		38	2.16	1.05	3.27	3.37	
No	48	1.52	− 0.18	3.22	5.86	0.8627	35	1.49	0.80	2.17	2.01	0.3005
Energy applied												
≤ 2000 J	19	0.47	− 0.02	0.97	1.02		13	1.15	0.14	2.17	1.68	
> 2000 J	73	1.71	0.50	2.93	5.20	0.0609	59	2.00	1.22	2.78	3.01	0.1734
Body mass index												
≤ 25 kg/m^2^	48	1.56	− 0.16	3.29	5.94		38	1.92	0.84	3.00	3.29	
> 25 kg/m^2^	37	1.27	0.38	2.17	2.68	0.7628	27	1.96	1.03	2.90	2.36	0.9525
Anesthesia												
Local anesthesia	78	0.96	0.47	1.46	2.19		59	1.75	1.02	2.47	2.80	−
Sedation	15	3.93	− 1.82	9.69	10.40	0.2885	14	2.21	0.53	3.90	2.91	0.5920

*PSS* proctological symptom score

**Fig. 6:**
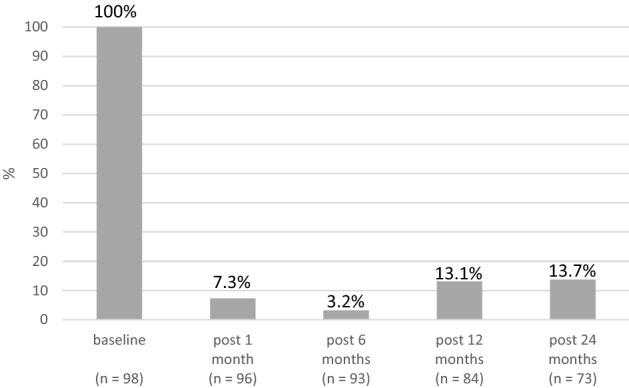
A graph showing the incidence of prolapse in %: test for binomial proportion = 1, *p* < 0.001

The haemorrhoidal prolapse decreased significantly 1, 6, 12 and 24 months (Fig. 6).

**Fig. 7:**
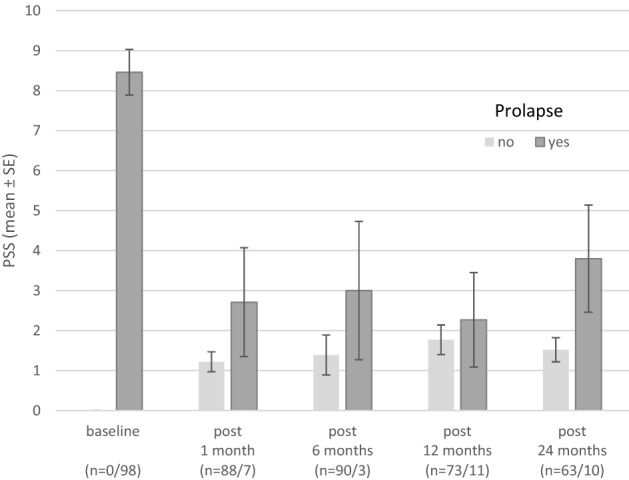
A graph showing PSS by presence of prolapse. No significant relation was shown: test for equal means (two group *t* test) was used: 1 months, *p* = 0.3167; 6 months, *p* = 0.4535; 12 months, *p* = 0.6892, 24 months, *p* = 0.1287. *PSS* proctological symptom scale

If haemorrhoidal prolapse recurred during follow-up, increased PSS values were seen, but this increase did not reach statistical significance (Fig. 7).

A total of 13 (12.7%) patients required repeat haemorrhoid therapy during the 2-year follow-up period. In 1 case, a surgical intervention was performed due to recurrence of prolapsing haemorrhoids and bleeding (haemorrhoidopexy using Longo’s procedure) 6 months after RFA. During follow-up, 12 patients received sclerotherapy and/or rubber band ligation for haemorrhoids due to bleeding (6 patients), soiling (4 patients) and itching (4 patients). No treatment was carried out if the patient was symptom-free, even if prolapsed haemorrhoidal tissue was seen again.

No other patient required any additional haemorrhoid treatment.

There were no cases of faecal incontinence following RFA.

## Discussion

RFA treatment with direct puncture is an innovative method of haemorrhoid treatment.

Our main observations were a significant decrease in overall proctological symptoms and for each single haemorrhoid symptom 1, 6, 12 and 24 months after the RFA. The same result was seen for haemorrhoid prolapse, which was significantly reduced during the two-year follow-up. Nineteen complications occurred after RFA, 8 of which were classified as major complications. One of the patients with major complications required surgical excision, the other complications were managed conservatively. Postoperative pain was low and 60% of the patients did not need any analgesic. Most patients (84%) returned to their normal daily activities the day after the procedure.

When subgroups of our study group (sex, previous treatment, BMI and age, type of anaesthesia) were analysed, there were no significant changes in PSS score within 2 years.

To our knowledge, the use of a needle to apply radiofrequency with direct puncture of the haemorrhoid through an anoscope was first published by our group [2]. This first experience with 102 patients showed an effective and safe treatment of haemorrhoids during a short follow-up period of 6 months. Several groups were able to reproduce the results in case series using the anoscope to expose the haemorrhoids [4–6]. One group [7] applied the radiofrequency by externalising the haemorrhoids to the anal verge, which requires general anaesthesia. Using the anoscope for RFA offers several advantages: the procedure can be performed under local anaesthesia in a proctology office without the need of an operation room. Moreover, the application of the radiofrequency to the proximal border of the haemorrhoid may result in thermally induced occlusion of the supplying haemorrhoid arteries, which can significantly help prevent recurrence of the haemorrhoid.

Patient satisfaction after surgical treatment of haemorrhoids is high when symptom control remains stable over the course of years. In the present study, the baseline PSS score had dropped significantly at the two-year follow-up. Prolapsing haemorrhoid tissue disappeared, a result that remained stable in 86.3% of patients after 2 years. Patients with recurrent prolapsed tissue had elevated PPS scores compared to patients without prolapse, thus underlining the recurring haemorrhoid disease.

Comparison of recurrence rates from the HubBLe study [8] show that haemorrhoids recurred in 49% of patients 1 year after rubber band ligature and in 30% of patients 1 year after haemorrhoidal artery ligature. In our study, haemorrhoid prolapse was detected in 13.1% of the patients 1 year and 13.7% of the patients 2 years after the radiofrequency treatment. A total of 13 (12.7%) patients required repeat haemorrhoid therapy within 2 years , which we considered as recurrence.

During follow-up, each patient underwent a complete proctological examination. Some patients were lost to follow-up for various reasons (15% after 1 year and 26% after 2 years). Most of the "lost patients" moved to another city and could not be contacted for follow-up.

In our study, we included only patients with third degree haemorrhoids in whom up to 2 haemorrhoidal segments were grade III, as we assumed that lower stages (I and II) can be adequately treated by sclerotherapy and rubber band ligation. In patients with more than 2 prolapsed bundles and in fourth degree haemorrhoids with external skin prolapse, our approach is surgical excision to remove both the haemorrhoids and the skin tag, which allows a normal anal verge after the wound heals. Eddema et al. [5] and Didelot et al. [7] included a small number of patients with grade four haemorrhoids in their case series and demonstrated that RFA can reduce haemorrhoid symptoms in these patients [5]. Available data show that RFA is also effective in grade two haemorrhoids [5, 6, 9]. Long-term follow-up of these patients is needed preferably in the form of a randomized trial against rubber band ligation.

From a technical point of view, radiofrequency ablation, performed with a small needle, causes coagulation of the haemorrhoidal tissue, followed by oedema formation, fibrosis and healing. There is no open wound, which reduces postoperative wound care and pain. In our series, 32 patients experienced no pain after the intervention, 7 patients had severe pain and the rest complained of only minor pain symptoms (Fig. 2). Interestingly, the average post-RFA VAS score was 2.5, which was significantly lower than after surgical excision (VAS score: 6.3–8.15) rubber band ligation and haemorrhoid artery ligation therapy [8, 10, 11]. Comparing the results of the VAS score with use of analgesics clearly favours the minimally invasive approach: about 60.2% of patients did not use analgesics (Fig. 3). This is in line with the results of other case series that found low postoperative pain after radiofrequency treatment [5, 12].

Bleeding after haemorrhoid treatment is a known complication and occurs in 0.6–2.8% after rubber band ligation [13, 14] and in 1.2–5% after haemorrhoid artery ligation [15, 16].

In our series, bleeding occurred in 6 cases during or after surgery and was the most common complication. Two cases were severe but stopped spontaneously without the need of surgical intervention. Drissi et al. reported six rectal bleeding episodes in 74 patients after RFA, of which two patients required surgical intervention [4]. Patients receiving anticoagulant therapy were more likely to experience postoperative bleeding (28%) than patients not receiving anticoagulant treatment (15%). In contrast, Eddema et al. and Didelot reported no or only minor bleeding in their series [5, 7]. In our series there was no correlation between the amount of the applied energy and the occurrence of postoperative bleeding (data not shown), which is an accordance with others [7].

One criterion for patient inclusion was the treatment of no more than two haemorrhoids per session. This decision was made to avoid increased pain, bleeding episodes or other postoperative disorders after the procedure. Again, no increased complication rate was observed in the patient groups when energy was elevated. This observation is consistent with randomized trials of rubber band ligation, in which triple rubber band ligations were as safe and effective as single band ligations [17, 18].

Therefore, it should be possible to extend RFA to more than two haemorrhoids without increasing the risk of side effects.

Seventeen patients requested a certificate of inability to work, with an average duration of 3.3 days. This result is remarkable compared to conventional surgical procedures such as excision treatment. The number of days after which patients returned to work following a haemorrhoid operation ranged from17.2 to 20.0 days in the case of Milligan-Morgan excision [10, 19–21].

The strengths of this study are the prospective data collection with clear statement of study objectives and the 2-year follow-up period. The bicentric design of the study with 5 participating surgeons allows generalization of the results in relation to the reality of care for third degree haemorrhoids. We only included grade III haemorrhoids to avoid ambiguity in patient selection. In addition, each follow-up was combined with a proctological examination, which ensured a view of the postoperative morphology and direct contact with the patient, when asking for residual haemorrhoid symptoms. Conversely there is a potential for bias, if the examiner is the same as the surgeon who performed the RFA. Another weakness of the study is, that we were only able to include 73 of 98 patients at the 2-year follow-up. 

 Although all surgeons were familiar with the RFA method before participating in the study, we cannot exclude a learning curve during the study period. Further studies on RFA should acknowledge the effect of a learning curve, as described by Pucher et al. for haemorrhoid ligation therapy [16]. For example, ulcerations and severe pain in our series were potentially attributed to a puncture of the RFA needle near to the sensitive anal channel, which was solved by a puncture clearly above the dentate line.

Finally, we did not assess faecal incontinence with a scoring system, but no case of faecal incontinence occurred after RFA in our series, as in the pilot study [2].

It is now essential to determine the value of this new method in the treatment of third-degree haemorrhoids with comparative studies, e.g., with surgical excision according to the Milligan-Morgan procedure and/or with rubber band ligature treatment. Future trials should also include a cost/effectiveness analysis.

## Conclusions

We found that RFA of third-degree haemorrhoids is safe and effective. The functional early and mid-term results seem competitive with the alternative haemorrhoid treatments. Because of the perioperative management with local anaesthesia in an outpatient setting, low postoperative pain and low number of complications, patient acceptance of this procedure is high.

## Data Availability

Data available on reasonable request.

## Availability of codes

Available on justified request.
